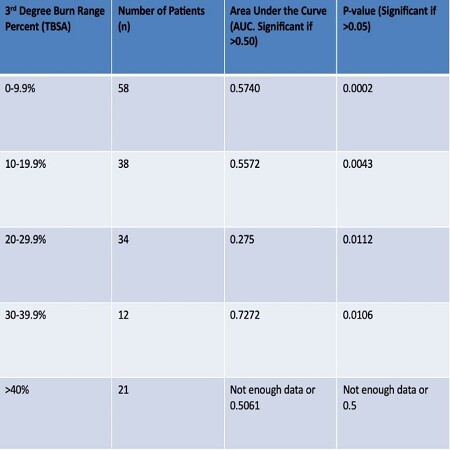# 794 Optimal Timing for Burn Surgery in Third-Degree Burns Is Three Days: A Retrospective Analysis

**DOI:** 10.1093/jbcr/irae036.335

**Published:** 2024-04-17

**Authors:** Amber Nanni, Caitlyn Matejka, Suyash Jain, Neeti Swami, Audra Zabava, Rebecca Gabrilska, Alan Pang, John A Griswold

**Affiliations:** Texas Tech University Health Sciences Center School of Medicine, Lubbock, TX; Texas Tech University Health Sciences Center School of Medicine, Royse City, TX; Texas Tech University Health Sciences Center, Lubbock, TX; Texas Tech University Health Sciences Center School of Medicine, Lubbock, TX; Texas Tech University Health Sciences Center School of Medicine, Royse City, TX; Texas Tech University Health Sciences Center, Lubbock, TX; Texas Tech University Health Sciences Center School of Medicine, Lubbock, TX; Texas Tech University Health Sciences Center School of Medicine, Royse City, TX; Texas Tech University Health Sciences Center, Lubbock, TX; Texas Tech University Health Sciences Center School of Medicine, Lubbock, TX; Texas Tech University Health Sciences Center School of Medicine, Royse City, TX; Texas Tech University Health Sciences Center, Lubbock, TX; Texas Tech University Health Sciences Center School of Medicine, Lubbock, TX; Texas Tech University Health Sciences Center School of Medicine, Royse City, TX; Texas Tech University Health Sciences Center, Lubbock, TX; Texas Tech University Health Sciences Center School of Medicine, Lubbock, TX; Texas Tech University Health Sciences Center School of Medicine, Royse City, TX; Texas Tech University Health Sciences Center, Lubbock, TX; Texas Tech University Health Sciences Center School of Medicine, Lubbock, TX; Texas Tech University Health Sciences Center School of Medicine, Royse City, TX; Texas Tech University Health Sciences Center, Lubbock, TX; Texas Tech University Health Sciences Center School of Medicine, Lubbock, TX; Texas Tech University Health Sciences Center School of Medicine, Royse City, TX; Texas Tech University Health Sciences Center, Lubbock, TX

## Abstract

**Introduction:**

Burn injuries pose a significant healthcare burden, with a substantial number of patients requiring hospital or emergency room treatment each year. Janzekovic generated renewed interest in early excision in 1970 when she reintroduced the concept of tangential excision of the necrotic tissue and immediate resurfacing with split-thickness skin grafts. Timely excision and grafting are now the standard surgical management of deep burns. However, existing studies lack comprehensive verification and fail to specify optimal operative periods for patients with third-degree burns. To address this gap, our objective was to determine the optimal skin graft operative days for burn surgery in patients suffering from third-degree burns.

**Methods:**

In this retrospective analysis, we isolated a group of burn patients who met the criteria for surgical burn repair, including burns injuries greater than 20% total body surface area (18-64 years old) or greater than 15% total body surface area (65-89 years old). The patients were categorized based on the percentage of third-degree burns, and an area under the curve (AUC) analysis was performed to evaluate the correlation between earlier surgical intervention and survival outcomes (AUC>0.5 with a p< 0.05 were considered significant.

**Results:**

A total of 163 patients fitting the criteria were included in the ten-year period 2012-2022. Our results demonstrated that earlier operative days were a significant predictor of mortality for patients with third-degree burn percentages within the ranges of 0.0-9.9% TBSA (n=58, AUC=0.5740, p=0.0002), 10-19.9% TBSA (n=38, AUC=0.5572, p=0.0043), and 30-39.9% TBSA (n=12, AUC=0.7272, p=0.0106). Interestingly, the operative day for patients with 20-29.9% TBSA burns did not significantly predict mortality (n=34, AUC=0.275, p=0.0112). Due to the limited number of patients with greater than 40% TBSA third-degree burns (n=21), further research with a larger sample size is required for accurate data analysis for that category.

**Conclusions:**

In summary, our retrospective analysis highlights the crucial role of operative timing in treating third-degree burns. We found a significant association between earlier surgical intervention and better survival outcomes for patients with burns covering less than 40% TBSA. While further research is needed for patients with greater than 40% TBSA burns, our findings provide valuable insights into optimizing surgical timing to enhance patient care and reduce mortality in burn management.

**Applicability of Research to Practice:**

Our research findings offer practical implications for healthcare practitioners. We emphasize the importance of timely surgical intervention for patients with third-degree burns covering less than 40% total body surface area (TBSA). Specifically, our study recommends performing skin graft surgery within three days of admission, when feasible for improved patient outcomes and reduced mortality rates in burn management.